# Impact of the COVID-19 pandemic on the oncologic activities (diagnosis, treatment, clinical trials enrollment) of a general hospital in a district with high prevalence of SARS-COV-2 in Italy

**DOI:** 10.1007/s00520-021-06667-y

**Published:** 2022-01-03

**Authors:** Massimo Ambroggi, Chiara Citterio, Stefano Vecchia, Alessandra Riva, Patrizia Mordenti, Luigi Cavanna

**Affiliations:** 1 Oncology Unit, Oncology and Hematology Department, Piacenza General Hospital, Via Taverna 49, 29121 Piacenza, Italy; 2 Pharmacy Unit, Piacenza General Hospital, Via Taverna 49, 29121 Piacenza, Italy

**Keywords:** COVID-19, Cancer management, Clinical studies, Oral cancer treatment, COVID-19, Cancer

## Abstract

**Purpose:**

Little is known about the real impact of the COVID-19 outbreak on the qualitative and quantitative fall-out on the management of cancer patients. Our objective was to provide evidence of the effects of SARS-COV-2 on the management of cancer patients in the real world.

**Methods:**

In a general hospital in a district in Italy with high prevalence of COVID-19 during the first wave, we retrospectively analyzed the data of oncologic activity, namely new cancer diagnosis, types of treatment (intravenous or by mouth), clinical research studies, and drug utilization, and compared the findings with those of 2019, before the pandemic. The data have been summarized in boxplot figures for median and interquartile range.

**Results:**

In 2020, a significant reduction in new cancer diagnosis was demonstrated when compared with 2019, with 17.4% fewer cancer diagnoses, 84.5% fewer patients enrolled in clinical trials, a 10.6% reduction in intravenous antitumor treatment, and a 42.7% increase in oral anticancer treatment.

**Conclusion:**

Our data indicate a significant reduction in cancer diagnosis, antitumor venous treatment, and patients enrolled in clinical research studies in 2020 compared with 2019, although there was a significant increase in oral treatment. These data suggest that the COVID-19 pandemic had a deep impact on the real-world management of cancer patients in a district of Italy with a high prevalence of COVID-19.

## Introduction

In December 2019, a new pathogen-enveloped RNA beta-coronavirus was identified that was later named severe acute respiratory syndrome coronavirus 2 (SARS-CoV-2). The virus causes severe disease in 14% of infected people [1, 2]. The World Health Organization (WHO) ruled coronavirus disease 2019 (COVID-19), caused by SARS-CoV-2, to be a public health emergency of international concern and declared a pandemic [3]. Italy was the second most exposed country worldwide, after China, during the first wave, with Lombardy, Emilia Romagna, and Veneto the regions most affected by COVID-19. The first Italian COVID-19 infection was reported in the Lombardy region on February 21, 2020. This first case was followed by an immediate and rapid rise in additional cases. The city of Piacenza (Emilia Romagna) is very close (12 km) to the epicenter of the outbreak of COVID-19, and the catastrophic nature of Lombardy’s outbreak has been widely publicized [4]. Clinical and pathological features of patients with COVID-19 have been reported, showing that SARS-CoV-2 infection causes clusters of severe and even fatal disease associated with admission to intensive care units (ICUs) and high mortality [5]. Cancer patients are at high risk of acquiring COVID-19 because of poor general health and their systemic immunosuppressive state caused by the cancer and/or anticancer treatments such as chemotherapy, radiation, surgery, and steroids. In addition, cancer patients frequently schedule visits to hospital and clinics, which can increase the risk of catching COVID-19 [6]. Patients with cancer have a markedly elevated risk of intubation, ICU admission, and death, both for those receiving active anticancer treatment and ones who have survived cancer [7]. In Italy’s oncological units, clinical consultations with patients who did not require active cancer treatment and follow-up were done at wider time intervals [8]. According to Italian oncologists, other types of preventive measures were established to reduce virus spread, such as subjecting patients to triage before hospital admission, and more specifically, using triage screening tools such as phone calls, virtual consultations, and telemedicine [9–11]. Furthermore, laboratory test diagnostics of SARS-CoV-2 infection, including nasopharyngeal swab RNA reverse transcriptase-polymerase chain reaction (RT-PCR) assay and rapid serological immunoassay, were implemented [12, 13].

We reported the first 25 cancer patients with COVID-19 complication [14], and 51 additional cases were subsequently described by our group [15], with a high mortality rate reported in these patient groups (36.00%, 25.49%, respectively) [14, 15]. It is well known that COVID-19 has presented many challenges to health-care providers and hospital systems. In this study, we investigated the role played by the COVID-19 outbreak on the management of cancer patients in terms of diagnosis, treatment, and oncologic clinical studies during this period. We compared oncologic activities, including the number of patients diagnosed and treated, as well as those enrolled in clinical studies and the type of treatment they received (intravenous, oral), between 2019 and the pandemic year of 2020.

## Materials and methods

We retrospectively evaluated the electronic databases of the oncological outpatients clinic of Piacenza General Hospital for the last two years (2019 and 2020) in order to evaluate the effect of SARS-COV-2 on the management of cancer patients (only patients with solid tumors), including diagnosis, treatment, and clinical research. We focused on three aspects: (1) new cancer diagnoses, (2) antineoplastic drugs and route of administration (intravenous or oral), and (3) patients enrolled in clinical trials (not for COVID-19).

All patients with a new diagnosis of cancer were scheduled and analyzed. All oral and intravenous antitumor treatments were recorded through an electronic database for centralized drug preparation of anticancer drugs. In addition, both randomized and nonrandomized clinical studies were analyzed and the differences reported. A protocol of proactive management of cancer patients based on telephone triage and restricted access to the hospital was implemented to enable the continuation of chemotherapy, biological treatments, targeted therapy, and immunotherapy. Beginning February 24, 2020, the following changes were introduced:

- Caregivers were not admitted to the hospital, except for when they accompanied non-self-sufficient outpatients.
- Patients were asked to wear respiratory masks and gloves.
- Body temperature was measured outside the hospital, and questionnaires asking for “flu-like” symptoms and possible COVID-19 contacts were completed.
- One day prior to a visit, telephone triage was performed to discuss with patients their clinical conditions and possible symptoms related to COVID-19, including cough, sore throat, fever, dyspnea, myalgia, diarrhea, nausea/vomiting, anosmia, and dysgeusia.
- One day prior to treatment administration, patients were given blood tests in separate rooms of the hospital to avoid crowding and allow the adequate organization of infusion treatments.
- Patients who complained of suspicious symptoms or had contact with COVID-19-positive people were asked to call their family physician for clinical evaluation or a designed number to seek appropriate medical support and not access the hospital for therapy.
- On the day of treatment administration, patients received additional clinical triage on hospital admission. This involved evaluation of respiratory tract symptoms, possible contact with COVID-19 positive persons, and fever check.
- All healthcare personnel began using personal protective equipment (PPE), including respiratory masks, caps, disposable overalls, and gloves, throughout the working day.

### Statistical analysis

Data have been summarized in boxplot figures for median and interquartile range. The boxes include the 25th, 50th, and 75th percentiles; the darker line indicates the median, and the whiskers indicate the range of the data, with spots indicating outliers. Comparisons were performed using the Mann–Whitney test. All tests were two-sided, and statistical significance was set at the 5% level. RStudio version 3.6.0 was used for the analysis and graphs. The research was conducted according to the principles of the Declaration of Helsinki and approved by the local institutional ethical committee.

## Results

**Fig. 1:**
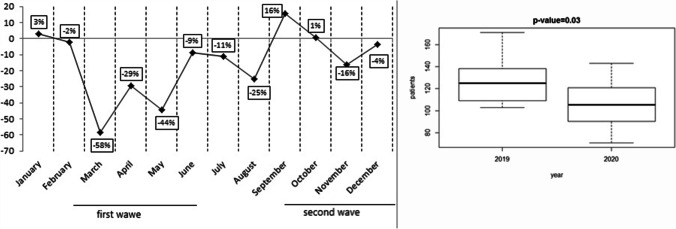
New diagnoses of malignant tumors (2020 vs 2019) and boxplot for median and interquartile range

**Fig. 2:**
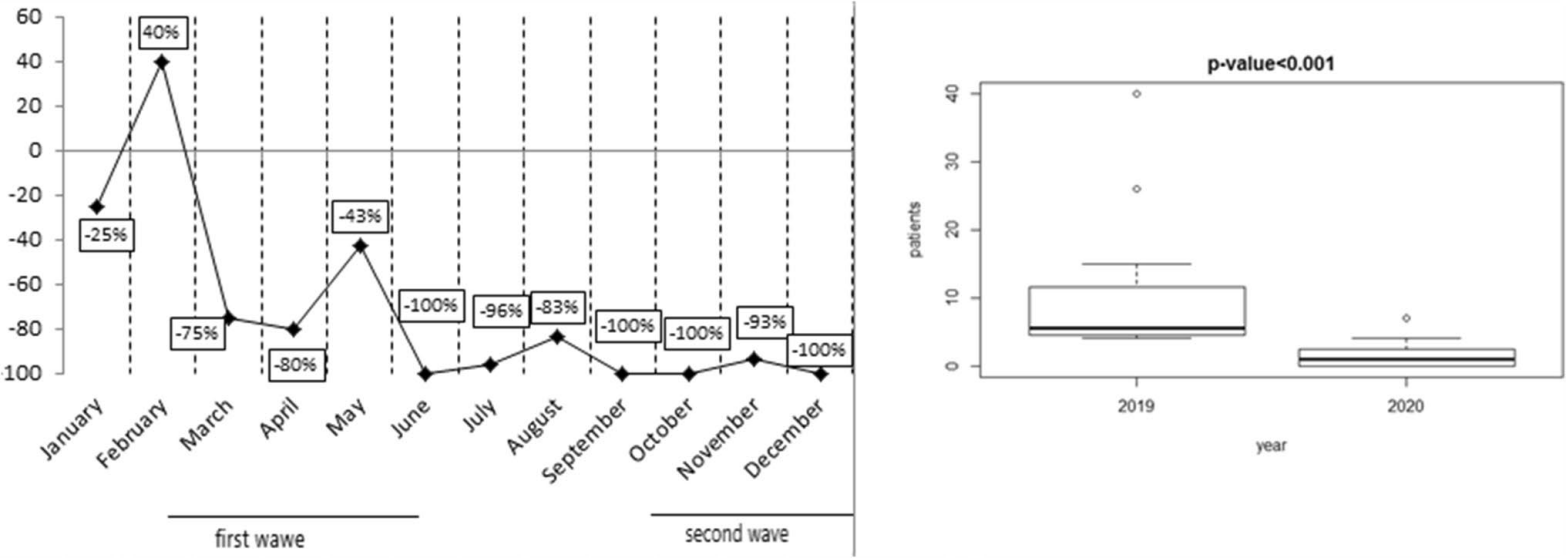
Patients enrolled in clinical trials (2020 vs 2019) and boxplot for median and interquartile range

**Fig. 3:**
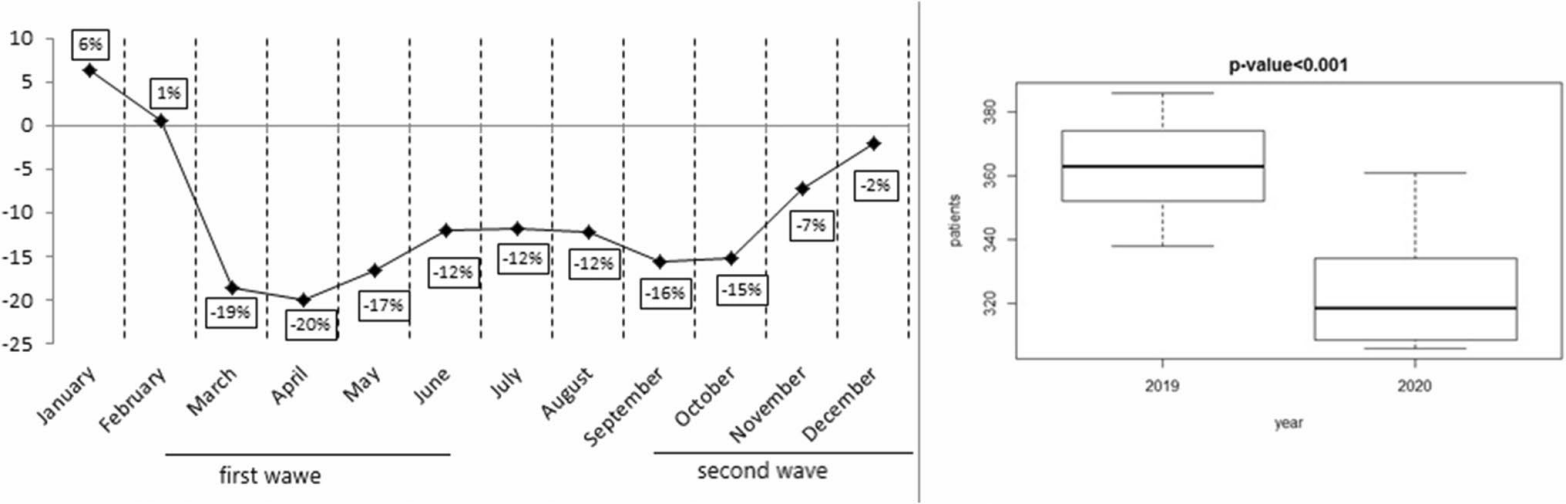
Oncologic intravenous treatment (2020 vs 2019) and boxplot for median and interquartile range

**Fig. 4:**
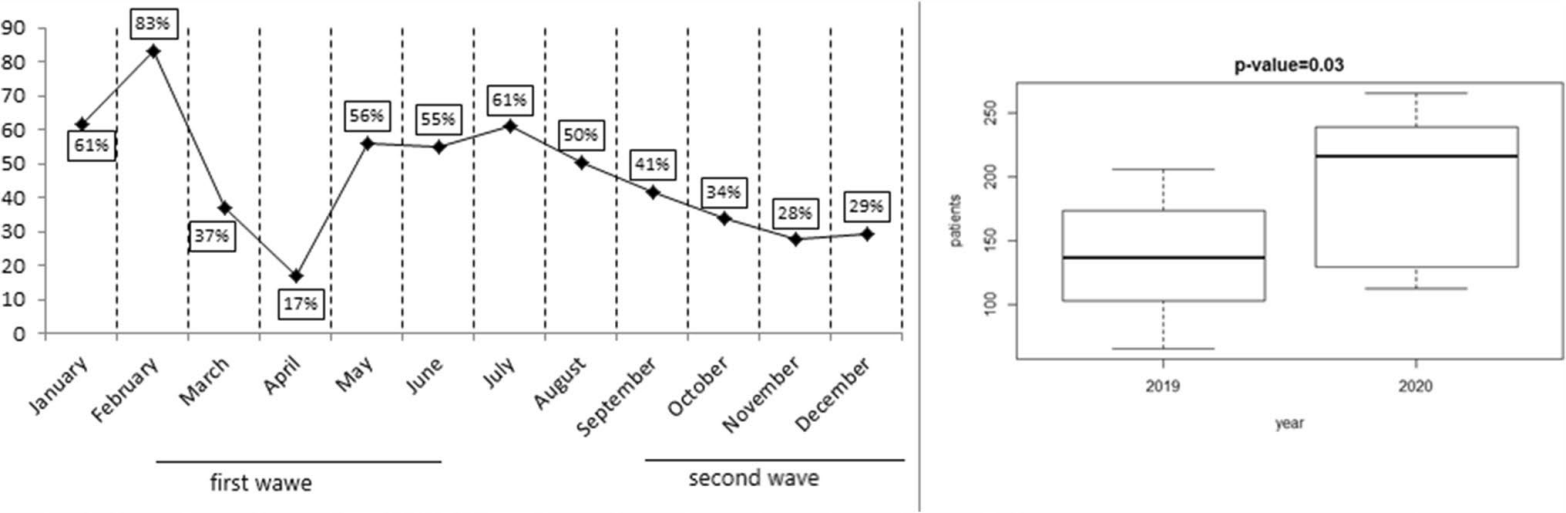
Oncologic oral treatment (2020 vs 2019) and boxplot for median and interquartile range

In the months of January and February, the number of new tumor diagnoses, patients enrolled in clinical trials, and patients treated with intravenously administered drugs was similar between 2019 and 2020 (Figs. 1, 2, and 3). The first case of COVID-19 was diagnosed in Italy on February 21, 2020. From March 2020, when SARS-COV-2 infection “exploded” in North Italy, fewer new cancer diagnoses were made for that year compared to 2019, although patients’ characteristics were similar in type and stage of cancer at diagnosis. Figure 1 shows the decrease, which was statistically significant (*p* = 0.003). Fewer patients enrolled in clinical trials, especially experimental ones and observational studies (*p* <0.001; Fig. 2), and were treated with drugs administered intravenously from March onwards, compared to the corresponding months of 2019 (*p* <0.001; Fig. 3). Considering new patients diagnosed with tumor, the difference between 2019 and 2020 from March to June was substantial. In March and May 2020, we observed the most significant reduction rate, compared to the same months of the previous year. From March 2020 to December 2020, more patients were treated with drugs administered orally (*p* = 0.03; Fig. 4), compared to the same period of 2019. When SARS-COV-2 infection improved (in summer 2020), the data also gradually improved; however, they did not achieve the same values as the corresponding period of 2019. The difference from 2019 and 2020 was seen also in October, November, and December, when SARS-COV-2 infection increased again in our district. In 2020, there was a 17.4% reduction in cancer diagnoses, 84.5% reduction in patients enrolled in clinical trials, 10.6% reduction in intravenous antitumor treatment, and a 42.7% increase in oral anticancer treatment compared to 2019.

## Discussion

Considering our single hospital experience from March 2020, we observed a decline in new cancer diagnoses, new clinical trial enrolment, and a change in how drugs were administered, with less intravenous and more oral drug administration. Comparing the months of January and February between 2019 and 2020, there was a similar number of cancer diagnoses, cancer treatments, and patients enrolled in clinical studies, and the data followed the trend of previous years. From March 2020, when SARS-COV-2 infection started to become severe in North Italy, and in particular in our city, the trend became statistically different. Fewer new patients were diagnosed with tumor or enrolled in clinical trials compared to 2019, fewer were treated with intravenous drugs, and more patients received oral drugs. What explanation can we find for this phenomenon? During the first wave of COVID-19, hospitals in North Italy faced a dramatic situation that had not been seen before, so that almost all of the doctors and nurses and all of the hospital’s facilities were dedicated to patients with SARS-COV-2 infection. In February 2020, oncologists in Piacenza first developed a strategic intervention to treat early patients with COVID-19 at home at the onset of symptoms, leaving them to remain at home with medical care and avoiding hospital admission as previously reported [16]. In our district, one of the territorial units of the Piacenza’s hospital became the first Italian hospital devoted entirely to COVID-19; however, there were so many patients affected with SARS-COV-2 infection that they were admitted to all hospitals and private clinics in the city of Piacenza and the surrounding province. Consequently, patients with other diseases found it difficult to visit hospitals and attend examinations. These included oncologic patients who, above all, were unable to receive surgical treatment, since medical oncology continued through oncologic therapy. In addition, patients would visit the hospital less frequently to avoid being infected by SARS-COV-2. Compared to the first wave, the second was less severe in Piacenza, and the number of deaths and hospitalizations was not comparable (approximately 15% of hospital’s beds in the second wave were taken up by COVID-19 patients, versus 95% of the beds during the first wave).

Despite COVID-19 representing a serious threat to public health, cancer still remains one of the main causes of death. Postponing or modifying treatment schedules may lead to worse outcomes, and it has a well-described impact on clinical outcomes. In addition, cancer patients may have a higher risk of infection due to frequent access to the hospital. For this reason, oncology associations quickly released guidelines on cancer care during the pandemic that recommended telemedicine, reducing medical evaluations, switching to subcutaneous or oral therapies when possible, and evaluating the benefits of each treatment [17]. As reported in our series, more patients in 2020 were treated with drugs administered orally. This meant that that they had to visit the hospital less frequently and spend less time there for therapies, needing only to be there when it was necessary and for intravenous treatment. In particular, a detailed guideline document was published on March 13, 2020, by the Italian Association of Medical Oncology (AIOM) [18]. On 20 March, 2020, the National Comprehensive Cancer Network published its recommendations for the management of cancer patients in endemic areas [19], and this was followed on 21 March, 2020, by a document by the European Society of Medical Oncology to support oncology professionals [20]. Based on these, the key interventions sought to (1) reduce hospital visits and provide telemedicine for follow-up visits, (2) delay medical tests and reserve radiological exams for patients with abnormal clinical findings, (3) enable telephone triage using a checklist to investigate suspicious clinical symptoms and trace contact with anyone having any symptoms of infection during the previous three weeks, (4) create a dedicated pathway for patients with symptoms or suspected contacts and have staff use PPE, and (5) modify the schedule and route of administration for patients with ongoing treatment according to the expected benefit of maintaining standard therapy. They also described modalities to manage surgical interventions and radiation therapy and to conduct clinical trials. Recent papers have investigated the impact of the COVID-19 pandemic on the attitudes and practices of Italian oncologists for breast cancer and related research activities [21, 22]. In particular, Poggio et al. [21] analyzed the results of a 29-question anonymous online survey that was sent by email to members of AIOM and the Italian Breast Cancer Study Group. The results describe changes in some oncologists’ attitudes and practices as a reasonable response to the health-care emergency (such as modifying weekly chemotherapy regimens to reduce patients’ hospital access or preferring oral therapies to be taken at home). However, some potentially alarming signs of undertreatment were observed, while clinical research and scientific activities were found to have reduced by 80.3% and 80.1%, respectively. Specific guidelines for the treatment of patients with gastrointestinal cancers [23] and older patients with cancer were also published [24]. Older cancer patients may have been denied supportive care because of their shorter life expectancy. The work provided special considerations to prevent the infection of older patients, namely separate scheduling to protect them from being infected, prompt activation of social services to ensure adequate medical supply, provision of food and daily transportation to cancer centers, close monitoring by phone, shorter courses of radiotherapy, and home telemedicine to avoid hospital admission. The results of a national survey showed that containment measures for oncologic patients had promptly been implemented throughout the whole country and in particular the use of protective devices and telemedicine, triage of patients accessing to the hospital, and delay of nonurgent visits [10]. A recent paper estimated the impact of delay in diagnosis on cancer survival outcomes for four tumor types in England and concluded that, due to the COVID-19 pandemic, an increase in the number of avoidable cancer deaths is expected in the UK [25]. In particular, a 7.9–9.6% increase in the number of deaths due to breast cancer up to 5 years after diagnosis, a 15.3–16.6% increase for colorectal cancer, a 4.8–5.3% increase for lung cancer, and a 5.8–6.0% increase for esophageal cancer are anticipated.

## Conclusion

It is well known that SARS-COV-2 can cause serious complications for most frail patients, including oncologic patients. In addition, the pandemic has severely affected the management of cancer patients, as reported here. In our department, the impact of COVID-19 was profound and had a deep impact on oncologic activities. In 2020, there was a 17.4% reduction in the number of cancer diagnoses, 84.5% reduction in patients enrolled in clinical trials, 10.6% reduction in intravenous antitumor treatment, and 42.7% increase in oral anticancer treatment, compared to 2019.

## Data Availability

The datasets generated and/or analyzed during the current study are available from the corresponding author on reasonable request.
